# Early Sepsis-Associated Acute Kidney Injury and Obesity

**DOI:** 10.1001/jamanetworkopen.2023.54923

**Published:** 2024-02-06

**Authors:** Yoon Hae Ahn, Si Mong Yoon, Jinwoo Lee, Sang-Min Lee, Dong Kyu Oh, Su Yeon Lee, Mi Hyeon Park, Chae-Man Lim, Hong Yeul Lee

**Affiliations:** 1 Department of Critical Care Medicine, Seoul National University Hospital, Seoul, Korea; 2 Division of Pulmonary and Critical Care Medicine, Department of Internal Medicine, Seoul National University Hospital, Seoul, Korea; 3 Department of Pulmonary and Critical Care Medicine, Asan Medical Center, Seoul, Korea

## Abstract

**Importance:**

The prevalence of obesity is increasing in the intensive care unit (ICU). Although obesity is a known risk factor for chronic kidney disease, its association with early sepsis-associated acute kidney injury (SA-AKI) and their combined association with patient outcomes warrant further investigation.

**Objective:**

To explore the association between obesity, early SA-AKI incidence, and clinical outcomes in patients with sepsis.

**Design, Setting, and Participants:**

This nationwide, prospective cohort study analyzed patients aged 19 years or older who had sepsis and were admitted to 20 tertiary hospital ICUs in Korea between September 1, 2019, and December 31, 2021. Patients with preexisting stage 3A to 5 chronic kidney disease and those with missing body mass index (BMI) values were excluded.

**Exposures:**

Sepsis and hospitalization in the ICU.

**Main Outcomes and Measures:**

The primary outcome was SA-AKI incidence within 48 hours of ICU admission, and secondary outcomes were mortality and clinical recovery (survival to discharge within 30 days). Patients were categorized by BMI (calculated as weight in kilograms divided by height in meters squared), and data were analyzed by logistic regression adjusted for key characteristics and clinical factors. Multivariable fractional polynomial regression models and restricted cubic spline models were used to analyze the clinical outcomes with BMI as a continuous variable.

**Results:**

Of the 4041 patients (median age, 73 years [IQR, 63-81 years]; 2349 [58.1%] male) included in the study, 1367 (33.8%) developed early SA-AKI. Obesity was associated with a higher incidence of SA-AKI compared with normal weight (adjusted odds ratio [AOR], 1.40; 95% CI, 1.15-1.70), as was every increase in BMI of 10 (OR, 1.75; 95% CI, 1.47-2.08). While obesity was associated with lower in-hospital mortality in patients without SA-AKI compared with their counterparts without obesity (ie, underweight, normal weight, overweight) (AOR, 0.72; 95% CI, 0.54-0.94), no difference in mortality was observed in those with SA-AKI (AOR, 0.85; 95% CI, 0.65-1.12). Although patients with obesity without SA-AKI had a greater likelihood of clinical recovery than their counterparts without obesity, clinical recovery was less likely among those with both obesity and SA-AKI.

**Conclusions and Relevance:**

In this cohort study of patients with sepsis, obesity was associated with a higher risk of early SA-AKI and the presence of SA-AKI modified the association of obesity with clinical outcomes.

## Key Points

**Question:**

In critically ill patients with sepsis, is obesity associated with incidence of early sepsis-associated acute kidney injury (SA-AKI)?

**Findings:**

In this cohort study of 4041 patients with sepsis, 33.8% developed early SA-AKI and patients with obesity had a higher risk of developing early SA-AKI.

**Meaning:**

These findings suggest that obesity is associated with early SA-AKI in patients in the intensive care unit.

## Teaser

This cohort study explores the association between obesity and sepsis-associated acute kidney injury and clinical outcomes among intensive care unit patients in Korea.

## Introduction

The prevalence of obesity is rising in intensive care units (ICUs) worldwide, and studies have reported that up to 20% of patients admitted to the ICU have obesity.^1,2,3^ Obesity is associated with numerous comorbidities, including diabetes, obstructive sleep apnea, and chronic kidney disease (CKD).^4,5^ Adipocytes contribute to the release of various adipokines,^6^ and this excess secretion of cytokines may lead to a low-grade systemic inflammatory state.^4,7^ In addition, elevated levels of free fatty acids and lipid intermediates contribute to insulin resistance, and chronic overactivity of the sympathetic nervous system can manifest as hypertension, leading to the development of metabolic derangements and comorbid diseases, such as congestive heart failure and stroke.^4^

Despite its association with chronic diseases, obesity is a heterogeneous condition that is often associated with paradoxical clinical outcomes. Previous studies have reported that obesity is associated with improved survival in patients with chronic heart failure,^8^ those receiving dialysis,^9^ and critically ill patients in the ICU.^10^ A meta-analysis of 22 studies conducted by Hogue et al^10^ showed that compared with patients with normal weight, patients with obesity had a lower risk of hospital mortality. This so-called obesity paradox can be explained by several factors. Patients with obesity have higher nutritional reserves that may contribute to improved survival,^11^ and high levels of cholesterol and lipoproteins can aid endotoxin removal.^4,12^ In patients with sepsis, high lipid levels can provide the necessary precursors for adrenal steroid synthesis.^10,13^ However, the exact mechanisms underlying the obesity paradox remain poorly understood, and the heterogeneous consequences of obesity make it difficult to predict clinical outcomes, especially in the context of critical illness.

Multiple studies have shown an association between obesity and the development of acute kidney injury (AKI) in patients with critical illness.^14,15,16^ Sepsis is the most common cause of AKI in critically ill patients,^17^ and sepsis-associated AKI (SA-AKI) is associated with poor clinical outcomes,^18^ including a higher risk of in-hospital mortality,^19,20^ longer hospital stays,^21^ and a greater chance of progression to CKD.^22^ Sepsis-associated AKI can be classified as either early or late. Whereas early SA-AKI is primarily caused by sepsis-induced kidney damage, late SA-AKI is usually an indirect consequence of sepsis treatment; early and late SA-AKI show phenotypic and prognostic differences.^23^ While obesity is a known risk factor for generalized AKI and CKD,^4^ its specific association with early SA-AKI remains unclear. As such, the primary objective of this study was to investigate the association between body mass index (BMI; calculated as weight in kilograms divided by height in meters squared) and early SA-AKI incidence in patients with sepsis.

Although the association of AKI with poor outcomes in patients with critical illness has been well documented,^17,24^ the clinical implications of AKI in patients with obesity are conflicting. While 1 study of 5232 ICU patients with severe AKI found the obesity paradox,^25^ another study showed no association between obesity and mortality in critically ill patients with postoperative AKI.^26^ Such conflicting evidence reflects the need for investigations on prognosis in patients with obesity and SA-AKI and a better understanding of whether the obesity paradox holds true in this particular subset of patients. Thus, the secondary objective of this study was to assess the association between BMI and clinical outcomes, including mortality and recovery, in critically ill patients with early SA-AKI.

## Methods

### Study Design and Patient Population

This nationwide, multicenter, prospective cohort study analyzed patients with sepsis in the Korean Sepsis Alliance registry between September 1, 2019, and December 31, 2021. Twenty tertiary or university-affiliated hospitals in South Korea that conduct educational programs on sepsis management participated in this study. A detailed description of the Korean Sepsis Alliance registry is provided in eMethods 1 in Supplement 1. Adult patients aged 19 years or older diagnosed with sepsis according to the Third International Consensus Definitions for Sepsis and Septic Shock (Sepsis-3) definitions^27^ and admitted to the ICU during the study period were included, and follow-up of clinical outcomes was conducted until hospital discharge or death. Any patients with preexisting stage 3A to 5 CKD and those with missing BMI values were excluded. All data were anonymized to ensure individual privacy, and the institutional review boards of all participating hospitals approved this study, including Seoul National University Hospital, where the work was performed. As this was an observational study, the decision to obtain or waive written informed consent was left to the discretion of the institutional review boards of the participating hospitals. This study was performed in line with the principles of the Declaration of Helsinki.^28^ The Strengthening the Reporting of Observational Studies in Epidemiology (STROBE) guideline was used to ensure the proper reporting of this cohort study.^29^

### Definitions and Outcome Measures

Based on the Acute Disease Quality Initiative 28 Workgroup’s consensus report,^23^ early SA-AKI was defined as the presence of sepsis criteria (as defined by Sepsis-3 criteria) and AKI criteria (as defined by Kidney Disease Improving Global Outcomes [KDIGO] criteria) within 48 hours of ICU admission. Late SA-AKI (SA-AKI occurring between 48 hours and 7 days of sepsis diagnosis) was not included in the analyses due to the unavailability of serum creatinine data beyond 48 hours. The primary outcome was the development of stage 1 to 3 early SA-AKI. The KDIGO classification of AKI used was as follows: stage 1, an absolute increase in serum creatinine level of 0.3 mg/dL or more (to convert to micromoles per liter, multiply by 88.4) or a greater than 1.5- to 2-fold increase from baseline; stage 2, a greater than 2- to 3-fold increase from baseline; and stage 3, a serum creatinine level of 4.0 mg/dL or more, a greater than 3-fold increase from baseline, or a requirement for kidney replacement therapy.^30^ The baseline creatinine level was defined as the serum creatinine concentration at sepsis diagnosis. As data on urine output were unavailable, only the serum creatinine criteria were used to define AKI. Secondary outcomes were clinical recovery within 30 days, ICU length of stay (LOS), hospital LOS, ICU mortality, in-hospital mortality, and discharge location. Clinical recovery was defined as survival to discharge within 30 days. The BMI was categorized into 4 groups based on the World Health Organization Asia-Pacific classification of weight by BMI, as follows: underweight (<18.5), normal weight (18.5-22.9), overweight (23-24.9), and obesity (≥25).^31^ Additional analyses were performed on the extreme ends of the BMI spectrum, including the severe underweight (<16.5) and severe obesity (≥30) categories.^31,32^

### Statistical Analysis

Categorical variables are expressed as counts and percentages, and continuous variables are reported as means and SDs or medians and IQRs. Descriptive statistics, including the Kruskal-Wallis test for numerical data and the χ^2^ test of independence for categorical variables, were used to compare baseline characteristics and outcome measures across BMI groups. The primary outcome was assessed using logistic regression analysis adjusted for key demographic and clinical factors associated with obesity, SA-AKI, or mortality (eMethods 2 in Supplement 1). Secondary outcomes were also analyzed using logistic regression analysis. Kaplan-Meier estimates were used to show the cumulative number of patients who experienced clinical recovery, and differences in recovery populations across BMI groups were assessed with the log-rank test. Multivariable fractional polynomial models analyzed the primary and secondary outcomes with BMI as a continuous variable, and restricted cubic spline curves assessed nonlinear associations between BMI and mortality. The results are presented as odds ratios (ORs) with corresponding 95% CIs. All analyses were 2-tailed, and *P* < .05 was considered to indicate statistical significance. A Bonferroni correction was used to account for multiple comparisons, and the significance thresholds were adjusted so that *P* < .05 divided by the number of comparisons was considered significant. Statistical analyses were performed using R, version 4.1.3 (R Foundation for Statistical Computing) and SPSS, version 27.0 for Windows (IBM).

## Results

### Study Participants

Among the 4889 patients with sepsis admitted to the ICU between September 1, 2019, and December 31, 2021, a total of 746 with preexisting CKD and 102 without BMI values were excluded. Of the patients admitted to the ICU during the study period, 4041 were included. Patients were categorized into 4 groups according to BMI as follows: 813 (20.1%), underweight; 1668 (41.3%), normal weight; 628 (15.5%), overweight; and 932 (23.1%), obesity (eFigure 1 in Supplement 1).

The baseline characteristics of the study population across the BMI groups are shown in Table 1 and eTable 1 in Supplement 1. Overall, the median BMI was 21.8 (IQR, 19.2-24.8), the median age was 73 years (IQR, 63-81 years); 1692 patients (41.9%) were female, and 2349 (58.1%) were male. In the cohort, 1834 patients (45.4%) required mechanical ventilation, 2294 (56.8%) presented with septic shock, and the median nonkidney sequential organ failure assessment score was 9 points (IQR, 6-11 points) on a scale of 0 to 20, with higher scores indicating worse organ dysfunction. The demographics of excluded patients with missing BMI data are shown in eTable 2 in Supplement 1.

**Table 1.** Baseline Characteristics Across Body Mass Index Groups

Variable	Participants^a^			
	Underweight (n = 813)	Normal weight (n = 1668)	Overweight (n = 628)	Obesity (n = 932)
Age, median (IQR), y	76 (64-83)	73 (63-81)	72 (63-80)	72 (61-79)
Sex				
Female	318 (39.1)	648 (38.8)	266 (42.4)	460 (49.4)
Male	495 (60.9)	1020 (61.2)	362 (57.6)	472 (50.6)
Body mass index, median (IQR)	16.8 (15.6-17.8)	20.9 (19.8-22.0)	23.9 (23.4-24.4)	27.1 (25.9-29.3)
Comorbidities				
Cardiovascular disease	148 (18.2)	353 (21.2)	130 (20.7)	193 (20.7)
Diabetes	227 (27.9)	577 (34.6)	228 (36.3)	368 (39.5)
Chronic lung disease	140 (17.2)	206 (12.4)	78 (12.4)	112 (12.0)
Chronic liver disease	57 (7.0)	171 (10.3)	67 (10.7)	100 (10.7)
Solid malignant tumor	228 (28.0)	533 (32.0)	191 (30.4)	270 (29.0)
Hematologic malignant tumor	37 (4.6)	107 (6.4)	62 (9.9)	71 (7.6)
Chronic neurologic disease	272 (33.5)	418 (25.1)	130 (20.7)	164 (17.6)
Charlson Comorbidity Index score, mean (SD)	5 (2)	5 (2)	5 (2)	5 (2)
Clinical frailty scale score, mean (SD)^b^	6 (2)	5 (2)	5 (2)	5 (2)
SAPS III score, median (IQR)^c^^,^^d^	71 (62-83)	72 (63-85)	72 (62-84)	72 (61-85)
SOFA score, median (IQR)^c^^,^^e^	9 (6-12)	9 (7-12)	10 (7-12)	10 (7-13)
Nonkidney SOFA score, median (IQR)^c^^,^^f^	8 (6-11)	9 (6-11)	9 (6-11)	9 (6-12)
Supportive care				
Vasopressors^c^	628 (77.2)	1334 (80.0)	482 (76.8)	729 (78.2)
Mechanical ventilation^c^	389 (47.8)	748 (44.8)	274 (43.6)	423 (45.4)
Transfusion^c^	143 (17.6)	401 (24.0)	148 (23.6)	213 (22.9)
Adjunctive corticosteroid therapy^c^	164 (20.2)	399 (23.9)	162 (25.8)	237 (25.4)
Septic shock^c^	434 (53.4)	955 (57.3)	357 (56.8)	548 (58.8)
Laboratory values, median (IQR)^c^				
WBC count, ×10^3^/μL	11.9 (6.4-18.1)	11.8 (6.4-18.0)	11.2 (4.8-16.7)	12.1 (6.3-18.4)
Hemoglobin level, g/dL	10.0 (8.7-11.5)	10.1 (8.6-11.8)	10.2 (8.7-11.9)	10.4 (8.8-12.3)
Platelet count, ×10^3^/μL	179 (98-262)	140 (71-222)	132 (69-204)	128 (66-214)
BUN level, mg/dL	28 (19-44)	29 (19-46)	30 (20-48)	29 (20-46)
Creatinine level, mg/dL	1.01 (0.65-1.68)	1.30 (0.83-2.08)	1.50 (0.95-2.33)	1.50 (0.99-2.38)
Albumin level, g/dL	2.7 (2.3-3.0)	2.7 (2.4-3.1)	2.8 (2.4-3.2)	2.8 (2.5-3.2)
Lactate level, mmol/L	2.5 (1.5-4.6)	2.8 (1.6-5.6)	2.8 (1.7-5.6)	2.9 (1.6-5.6)
CRP level, mg/dL	13.1 (6.5-21.2)	13.8 (6.4-22.0)	15.0 (6.8-23.4)	14.7 (6.7-24.1)
Type of infection				
Community acquired	485 (59.7)	1002 (60.1)	380 (60.5)	562 (60.3)
Nosocomial	328 (40.3)	666 (39.9)	248 (39.5)	370 (39.7)
Multidrug-resistant organisms^c^	241 (29.6)	439 (26.3)	153 (24.4)	228 (24.5)
Nephrotoxic antimicrobials^c^^,^^g^	153 (18.8)	411 (24.6)	165 (26.3)	260 (27.9)
Adequate antimicrobial therapy^c^^,^^h^	706 (86.8)	1450 (86.9)	545 (86.8)	834 (89.5)

Abbreviations: BUN, blood urea nitrogen; CRP, C-reactive protein; SAPS III, Simplified Acute Physiology Score III; SOFA, sequential organ failure assessment; WBC, white blood cell.

SI conversion: To convert albumin to g/L, multiply by 10; BUN to mmol/L, multiply by 0.357; creatinine to μmol/L, multiply by 88.4; CRP to mg/L, multiply by 10; hemoglobin to d/L, multiply by 10; lactate to mg/dL, divide by 0.111; platelet count to ×10^9^/L, multiply by 1.0; and WBC count to ×10^9^/L, multiply by 0.001.

^a^ Data are presented as number (percentage) of participants unless otherwise indicated. Body mass index was calculated as weight in kilograms divided by height in meters squared and was categorized into 4 groups based on the World Health Organization Asia-Pacific classification as follows: underweight (<18.5), normal weight (18.5-22.9), overweight (23-24.9), and obese (≥25).^31^

^b^ The clinical frailty scale score ranges from 1-9 points, with higher scores indicating increased frailty.

^c^ On the day of intensive care unit admission.

^d^ The SAPS III score ranges from 0-217 points, with higher scores indicating greater severity of acute illness.

^e^ The SOFA score ranges from 0-24 points, with higher scores indicating worse organ dysfunction.

^f^ The nonkidney SOFA score ranges from 0-20 points, with higher scores indicating worse organ dysfunction.

^g^ Nephrotoxic antimicrobials included glycopeptides, aminoglycosides, amphotericin B, or colistin.

^h^ Adequate empirical antimicrobial therapy was defined as the use of antibiotic agents with in vitro activity against suspected pathogens.

### Primary Outcome

**Figure 1.**
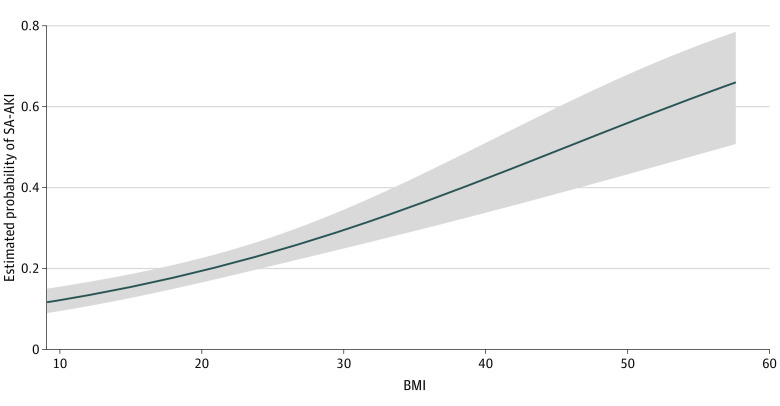
Estimated Probability of Early Sepsis-Associated Acute Kidney Injury (SA-AKI) Development Shaded area represents 95% CIs. BMI indicates body mass index (calculated as weight in kilograms divided by height in meters squared).

Of the 4041 patients included in the study, a total of 1367 (33.8%) developed early SA-AKI and 896 (22.2%) developed stage 3 early SA-AKI. Obesity was associated with a greater incidence of early SA-AKI compared with normal weight (adjusted OR [AOR], 1.40; 95% CI, 1.15-1.70), and higher BMI was associated with AKI severity, with stage 3 SA-AKI occurring in 263 patients (28.2%) in the group with obesity (AOR, 1.56; 95% CI, 1.25-1.95) (Table 2). When patients were grouped into 6 categories to include the severe underweight and severe obesity classifications, severe obesity was associated with higher incidence of both overall (AOR, 2.20; 95% CI, 1.53-3.15) and stage 3 (AOR, 2.37; 95% CI, 1.60-3.53) early SA-AKI (eTable 3 in Supplement 1). Multivariable fractional polynomial regression models with a continuous BMI scale showed similar results. Every increase in BMI of 10 was associated with a higher risk of early SA-AKI (OR, 1.75; 95% CI, 1.47-2.08) and stage 3 early SA-AKI (OR, 1.96; 95% CI, 1.60-2.40) (eTable 4 in Supplement 1). The estimated probabilities of overall and stage 3 early SA-AKI development also increased linearly with increasing BMI (Figure 1 and eFigure 2 in Supplement 1, respectively).

**Table 2.** Early Sepsis-Associated Acute Kidney Injury Incidence According to Body Mass Index^a^

Outcome	Underweight (n = 813)	Normal weight (n = 1668)	Overweight (n = 628)	Obesity (n = 932)
Stage 1, 2, or 3 SA-AKI				
Participants, No. (%)	201 (24.7)	546 (32.7)	242 (38.5)	378 (40.6)
Adjusted OR (95% CI)^b^	0.71 (0.57-0.89)^c^	1 [Reference]	1.33 (1.07-1.66)^c^	1.40 (1.15-1.70)^c^
Stage 3 SA-AKI				
Participants, No. (%)	125 (15.4)	342 (20.5)	166 (26.4)	263 (28.2)
Adjusted OR (95% CI)^b^	0.78 (0.60-1.01)	1 [Reference]	1.49 (1.16-1.91)^c^	1.56 (1.25-1.95)^c^

Abbreviations: OR, odds ratio; SA-AKI, sepsis-associated acute kidney injury.

^a^ Body mass index was calculated as weight in kilograms divided by height in meters squared and was categorized into 4 groups based on the World Health Organization Asia-Pacific classification as follows: underweight (<18.5), normal weight (18.5-22.9), overweight (23-24.9), and obese (≥25).^31^

^b^ Adjusted for age; sex; comorbidities; Charlson Comorbidity Index score; clinical frailty scale; Simplified Acute Physiology Score III; nonkidney sequential organ failure assessment score; mechanical ventilation; transfusion; septic shock; hemoglobin level; lactate level; albumin level; C-reactive protein level; use of vasopressors, corticosteroids, or nephrotoxic antimicrobials; primary site of infection; and type of infection.

^c^ Statistically significant after Bonferroni correction (*P* < .017) compared with the group with normal weight.

### Secondary Outcomes

**Figure 2.**
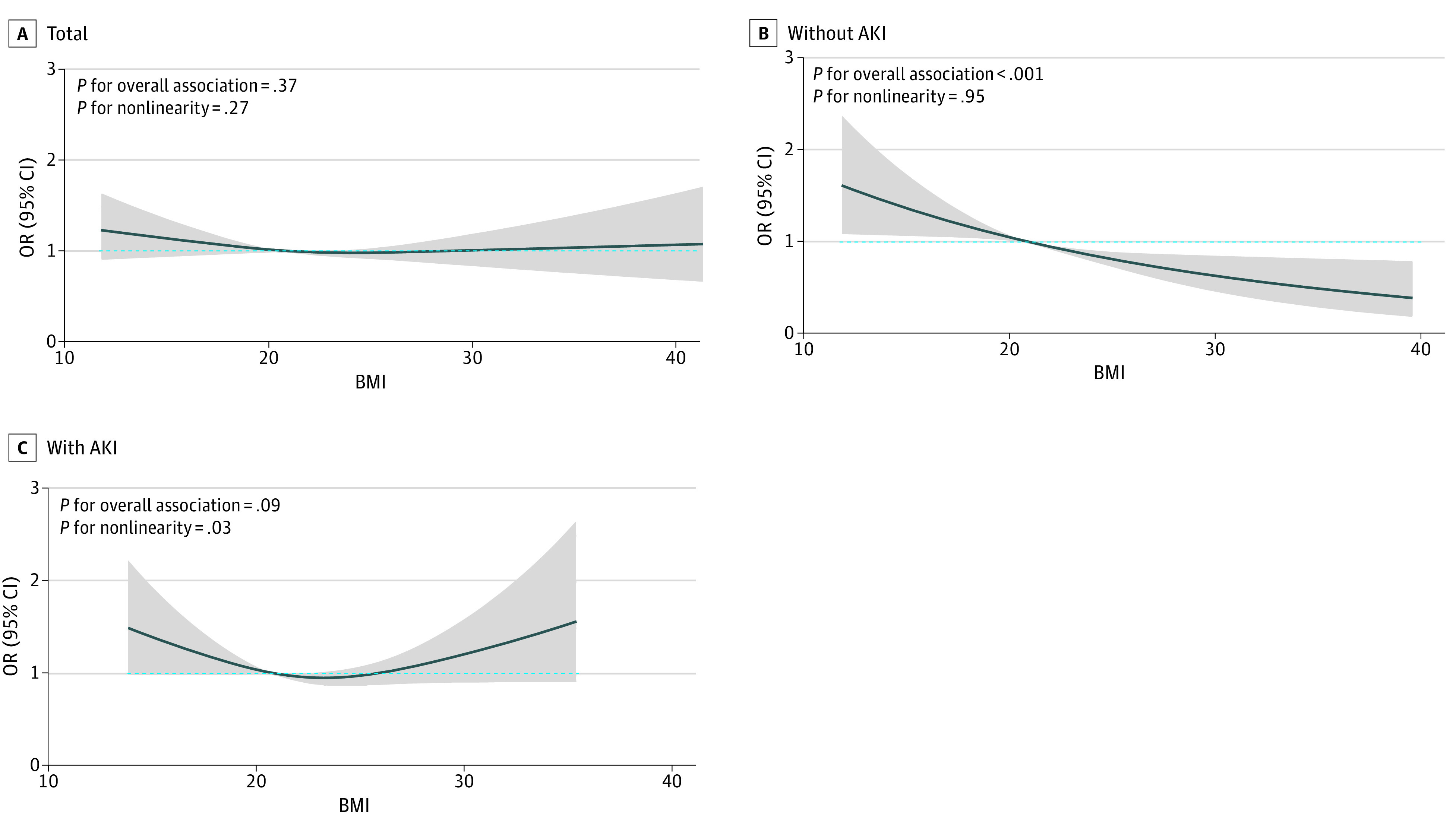
Association Between Body Mass Index (BMI) and In-Hospital Mortality Shaded area represents 95% CIs, and the median BMI (calculated as weight in kilograms divided by height in meters squared) in the group with normal weight (20.9) was the reference standard, as indicated by the dashed blue line. AKI indicates acute kidney injury; OR, odds ratio.

The secondary outcomes of clinical recovery within 30 days, ICU LOS, hospital LOS, ICU mortality, and in-hospital mortality across the BMI groups are summarized in eTables 5 and 6 in Supplement 1. While patients with a higher BMI were more likely to be discharged to home rather than to a stepdown facility, no other significant differences in terms of clinical recovery, ICU LOS, hospital LOS, ICU mortality, and in-hospital mortality were observed across BMI groups (eTable 5 in Supplement 1). Secondary outcomes were further analyzed according to the presence of early SA-AKI. While patients with obesity without SA-AKI paradoxically showed improved in-hospital mortality compared with their counterparts without obesity (AOR, 0.72; 95% CI, 0.54-0.94), those with SA-AKI did not (AOR, 0.85; 95% CI, 0.65-1.12). Similar results were observed when BMI was used as a continuous variable. Restricted cubic spline curves showed that while obesity was associated with improved in-hospital mortality in patients without SA-AKI (Figure 2B), no survival advantages were seen in those with SA-AKI (Figure 2C). The association among BMI, SA-AKI, and ICU mortality are shown in eFigure 3 in Supplement 1, and the results of the polynomial regression analyses are shown in eTable 7 in Supplement 1.

**Figure 3.**
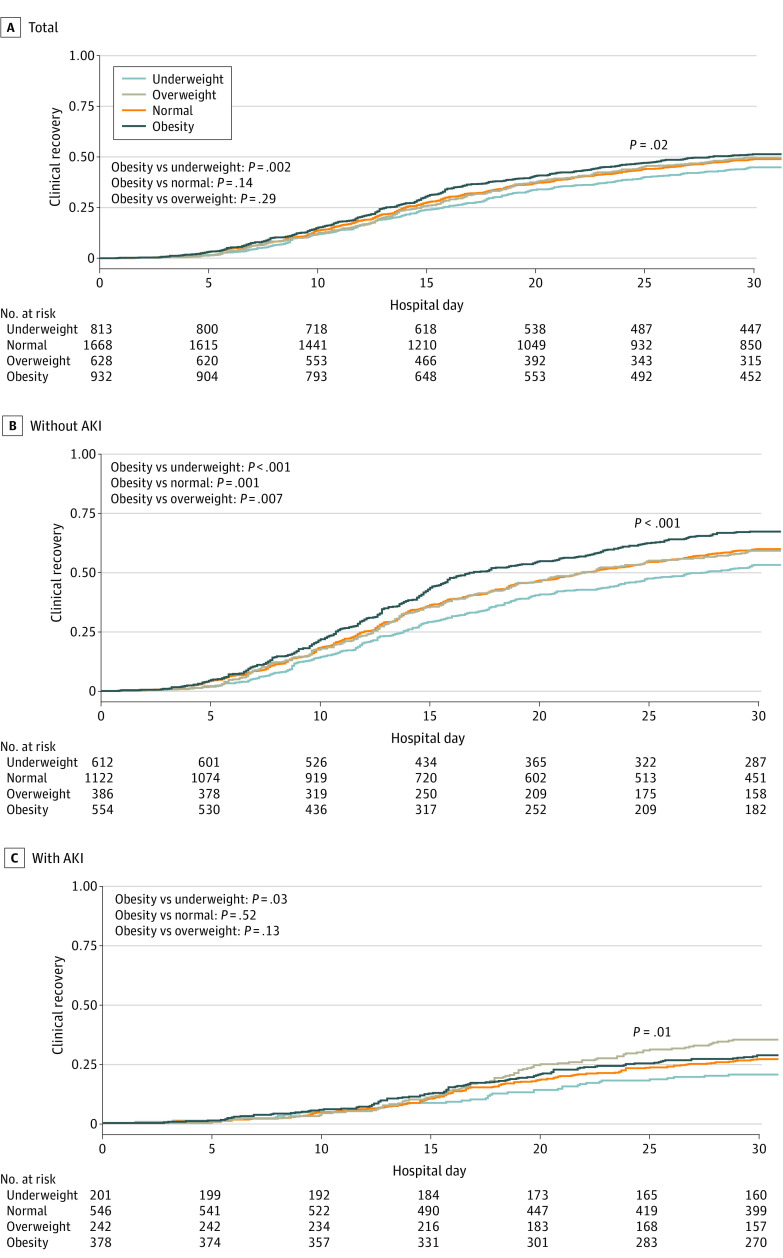
Clinical Recovery Within 30 Days According to Early Sepsis-Associated Acute Kidney Injury (AKI) Status Clinical recovery was defined as survival to discharge within 30 days.

In addition, the presence of early SA-AKI was associated with higher ICU and in-hospital mortality within each BMI category (eTable 8 in Supplement 1). The Kaplan-Meier estimates of the cumulative number of patients who made a clinical recovery within 30 days, stratified according to BMI, are shown in Figure 3. Although patients with obesity without SA-AKI were more likely to experience clinical recovery than their counterparts without obesity (Figure 3B), patients with obesity and SA-AKI were less likely to experience clinical recovery (Figure 3C). The primary and secondary outcomes in the full cohort, including the excluded patients with CKD, are shown in eTable 9 in Supplement 1.

## Discussion

This nationwide, multicenter, prospective cohort study of critically ill patients with sepsis showed that obesity was significantly associated with development of early SA-AKI, and the presence of SA-AKI was significantly associated with a higher risk of mortality across all BMI groups. While increasing BMI was associated with improved survival and a higher probability of clinical recovery in patients without SA-AKI, the protective benefits of obesity were attenuated in those with SA-AKI. As more patients with obesity are admitted to the ICU every year, it is important to have a better understanding of the clinical implications of obesity on patient outcomes in the context of critical illness. To this extent, our study showed an association among obesity, early SA-AKI, and clinical outcomes in critically ill patients with sepsis.

Our study showed that increasing BMI was associated with AKI, as previously described in the literature.^14,33^ In a study including ICU patients, each increase in BMI of 5 was associated with a 10% increased risk of developing AKI.^15^ Another retrospective cohort study of 773 patients showed that obesity was significantly associated with an increased risk of AKI (OR, 2.70; 95% CI, 1.01-7.26).^16^ The association between BMI and the development of AKI can be attributed to multiple factors. Patients with obesity show alterations in intrarenal hemodynamics, in which glomerular hyperfiltration and increased tubular salt reabsorption result in systemic hypertension and nephron mass reduction.^34^ Adipose tissue stimulates the production of angiotensinogen and aldosterone, thereby activating the renin-angiotensin-aldosterone system; enhanced activation of the renin-angiotensin-aldosterone system is likely to be correlated with the development of proteinuric kidney injury observed in obesity.^34^ In addition, increased secretion of cytokines and hormones from adipocytes may contribute to inflammation and endothelial cell activation, rendering the host kidneys more susceptible to injury.^35,36^ Finally, patients with obesity in the ICU are at a higher risk of intra-abdominal hypertension, which may contribute to kidney dysfunction through a combination of venous congestion and poor arterial perfusion.^37^ As SA-AKI is associated with increased mortality in patients with sepsis,^38^ recognizing factors associated with SA-AKI, including obesity, is a necessary step toward early intervention and improvement of patient outcomes.

The protective benefits of obesity during critical illness have been investigated in previous studies.^10,39^ Prescott et al^40^ observed that patients with higher BMI who were hospitalized with severe sepsis experienced lower 1-year mortality than patients with normal weight (OR, 0.59; 95% CI, 0.39-0.88). Another retrospective cohort study of 1 763 000 patients with sepsis showed that obesity was associated with a 16% decrease in the odds of mortality,^41^ albeit not all patients included in the 2 studies were sufficiently ill to be admitted to the ICU. Although our findings showed that obesity was associated with lower mortality in patients without SA-AKI, the paradoxical advantages of obesity were not evident in those with SA-AKI. Our results suggest that the presence of SA-AKI may offset the potential benefits of obesity in critically ill patients.

The development of AKI in critically ill patients with sepsis has been associated with high mortality.^18,42^ A prospective cohort study of 1177 patients with sepsis admitted to 198 ICUs across Europe reported a mortality rate of up to 41% in patients with AKI.^43^ Danziger et al^15^ previously demonstrated an association between AKI and increased mortality in a cohort of 15 000 critically ill patients. Consistent with previous studies, our study also showed that the development of early SA-AKI was associated with higher mortality in all BMI groups. While higher BMI was associated with better clinical recovery in patients without SA-AKI, no such advantage in recovery was seen in those with SA-AKI. Thus, the presence of SA-AKI may be indicative of higher illness severity, which may explain why the obesity paradox was not observed in those with early SA-AKI. Research has shown that AKI has the potential to cause systemic complications,^20^ and a previous study of 7967 patients with sepsis showed that the obesity paradox was only evident in those with lower illness severity.^44^ Similarly, a study conducted by Pedersen et al^26^ reported that no difference in mortality was found between patients with obesity and AKI and those with normal weight and AKI. As early SA-AKI is associated with a poor prognosis,^45^ prompt initiation of adequate kidney protective measures may be needed to improve patient outcomes.

The results of our study showed that early SA-AKI modified the association between obesity and clinical outcomes, including mortality and recovery from critical illness. Animal models have shown that obesity is conducive to inflammation, and heightened inflammation during sepsis can potentially lead to adverse outcomes.^46^ A swine model of endotoxic shock showed a significantly higher degree of circulatory compromise in swine with obesity compared with controls with normal weight, resulting in a worse state of organ failure with increased levels of proinflammatory cytokines.^46,47^ Another model of swine with obesity showed that obesity was associated with increased oxidative stress and reduced endogenous nitric oxide production.^47,48^ Such mechanisms of hyperinflammation may outweigh any potential benefits of obesity during critical illness, especially in the context of SA-AKI, in which the combined effects of inflammation and oxidative stress play a decisive role in its pathogenesis.^49^ While the exact pathophysiologic mechanisms behind the association among obesity, AKI, and mortality in patients with critical illness remain obscure, our study showed that patients with obesity had higher risk of developing SA-AKI and that the presence of SA-AKI and its multisystem complications were associated with patient outcomes.

### Strengths and Limitations

Our study has several strengths. This nationwide prospective study included a relatively large number of critically ill patients with sepsis. While the majority of the existing literature included a broad population of critically ill patients, we were able to perform an in-depth investigation of the association between early SA-AKI and obesity in a specific subpopulation of patients with sepsis. Our results showed that obesity was significantly associated with development of early SA-AKI and that the presence of SA-AKI modified the association of obesity with outcomes in patients with critical illness. To our knowledge, our study is the first to show differences in clinical outcomes according to the presence of early SA-AKI. While increasing BMI was associated with better survival and clinical recovery in patients without SA-AKI, no differences in outcomes were seen in patients with SA-AKI.

Our study also has some limitations. First, although we adjusted for several potential confounders in our regression analysis, the risk of unmeasured confounders may still exist. Second, the use of BMI as a surrogate marker for obesity is arbitrary; BMI alone does not consider body composition, including fat distribution and muscle mass. Third, there was a potential for measurement error in the evaluation of the Sepsis-3 criteria, as the assessment process was left to the individual judgment of the physicians at the participating centers, and albeit for a small proportion of cases, the lack of laboratory values needed to determine the initial sequential organ failure assessment scores may have led to an underestimation of sepsis incidence. Fourth, AKI was defined solely based on creatinine measurements because of the unavailability of data on daily urine output. Studies have shown that disregarding the urine output criteria reduces diagnostic sensitivity and can underestimate the true incidence of AKI.^50,51^ However, the majority of the false-negative cases were stage 1 AKI events, and Allen et al^50^ reported that incorporating the urine output criteria had little influence on the incidence of stage 2 and 3 AKI. Moreover, the dynamic and complex nature of critical illness inevitably introduces confounding factors, such as fluid resuscitation, diuretic use, and hemodynamic disturbances, that can contribute to urine output regardless of intrinsic kidney function.^52^ Although we recognize that using the serum creatinine criteria alone to define AKI may have underestimated the overall incidence of early SA-AKI and limited the generalizability of our findings, we believe that the majority of the false-negative cases were stage 1 early SA-AKI events, which have little clinical significance. Fifth, we were unable to determine the incidence and clinical outcomes of late SA-AKI, as data on serum creatinine levels beyond 48 hours were unavailable. Sixth, although we showed the short-term implications of early SA-AKI in patients across 4 different BMI categories, we were unable to investigate its long-term consequences following recovery from critical illness. Further studies on long-term outcomes, including the risk of progression to CKD, are needed.

## Conclusions

In this cohort study of critically ill patients with sepsis, those with a higher BMI had a higher risk of developing early SA-AKI. While the paradox of improved survival and higher probability of clinical recovery was apparent in patients with obesity without SA-AKI, we found that the presence of SA-AKI modified the association of obesity with clinical outcomes. Our findings suggest that obesity is associated with early SA-AKI in patients in the ICU and highlight the need for future research on the mechanisms underlying the complex association among obesity, SA-AKI, and clinical outcomes in patients with sepsis.

## Notes

Supplement 1. **eMethods 1.** Description of the Korean Sepsis Alliance Registry **eMethods 2.** Baseline Clinical Factors Adjusted in the Main Analysis **eFigure 1.** Study Design **eFigure 2.** The Predicted Probability of Stage 3 Early SA-AKI **eFigure 3.** Association Between Body Mass Index and ICU Mortality **eTable 1.** Baseline Characteristics Across Body Mass Index Groups **eTable 2.** Baseline Characteristics of Patient With Missing Body Mass Index Data **eTable 3.** Early SA-AKI Incidence According to Body Mass Index, Including Severe Underweight and Obese Categories **eTable 4.** Summary of Multivariable Fractional Polynomial Transformations Applied to the Primary Outcome **eTable 5.** Clinical Outcome of Early SA-AKI According to Body Mass Index **eTable 6.** Clinical Outcomes According to Body Mass Index, Including Severe Underweight and Obese Categories **eTable 7.** Summary of Multivariate Fractional Polynomial Transformations Applied to the Secondary Outcome of In-Hospital Mortality **eTable 8.** ICU and In-Hospital Mortality Within Each Body Mass Index Category According to Early SA-AKI Status **eTable 9.** Early SA-AKI Incidence and Clinical Outcomes of the Full Cohort, Including Excluded Patients With CKD

Supplement 2.
Korean Sepsis Alliance Investigators

Supplement 3.
Data Sharing Statement
