# Supplementary material for: Early Sepsis-Associated Acute Kidney Injury and Obesity
Source: JAMA Netw Open. 2024 Feb 6;7(2):e2354923. doi: 10.1001/jamanetworkopen.2023.54923 (PMC10848068; doi:10.1001/jamanetworkopen.2023.54923)
Supplement: Supplement 1. — eMethods 1. Description of the Korean Sepsis Alliance Registry eMethods 2. Baseline Clinical Factors Adjusted in the Main Analysis eFigure 1. Study Design eFigure 2. The Predicted Probability of Stage 3 Early SA-AKI eFigure 3. Association Between Body Mass Index and ICU Mortality eTable 1. Baseline Characteristics Across Body Mass Index Groups eTable 2. Baseline Characteristics of Patient With Missing Body Mass Index Data eTable 3. Early SA-AKI Incidence According to Body Mass Index, Including Severe Underweight and Obese Categories eTable 4. Summary of Multivariable Fractional Polynomial Transformations Applied to the Primary Outcome eTable 5. Clinical Outcome of Early SA-AKI According to Body Mass Index eTable 6. Clinical Outcomes According to Body Mass Index, Including Severe Underweight and Obese Categories eTable 7. Summary of Multivariate Fractional Polynomial Transformations Applied to the Secondary Outcome of In-Hospital Mortality eTable 8. ICU and In-Hospital Mortality Within Each Body Mass Index Category According to Early SA-AKI Status eTable 9. Early SA-AKI Incidence and Clinical Outcomes of the Full Cohort, Including Excluded Patients With CKD [file jamanetwopen-e2354923-s001.pdf]

Supplemental Online Content

Ahn YH, Yoon SM, Lee J, et al. Early sepsis-associated acute kidney injury and obesity. *JAMA Netw Open*. 2024;7(2):e2354923. doi:10.1001/jamanetworkopen.2023.54923

eMethods 1. Description of the Korean Sepsis Alliance Registry

eMethods 2. Baseline Clinical Factors Adjusted in the Main Analysis

eFigure 1. Study Design

eFigure 2. The Predicted Probability of Stage 3 Early SA-AKI

eFigure 3. Association Between Body Mass Index and ICU Mortality

eTable 1. Baseline Characteristics Across Body Mass Index Groups

eTable 2. Baseline Characteristics of Patient With Missing Body Mass Index Data

eTable 3. Early SA-AKI Incidence According to Body Mass Index, Including Severe Underweight and Obese Categories

eTable 4. Summary of Multivariable Fractional Polynomial Transformations Applied to the Primary Outcome

eTable 5. Clinical Outcome of Early SA-AKI According to Body Mass Index

eTable 6. Clinical Outcomes According to Body Mass Index, Including Severe Underweight and Obese Categories

eTable 7. Summary of Multivariable Fractional Polynomial Transformations Applied to the Secondary Outcome of In-Hospital Mortality

eTable 8. ICU and In-Hospital Mortality Within Each Body Mass Index Category According to Early SA-AKI Status

eTable 9. Early SA-AKI Incidence and Clinical Outcomes of the Full Cohort, Including Excluded Patients With CKD

This supplemental material has been provided by the authors to give readers additional information about their work.

© 2024 Ahn YH et al. *JAMA Network Open.*

eMethods 1. Description of the Korean Sepsis Alliance Registry

All hospitals were required to report prospectively collected data for patients with sepsis using a web-based database system (http://sepsis.crf.kr/) that included demographics, comorbidities, illness severity, sepsis treatment, laboratory variables, ICU resource utilization, and clinical outcomes. Review of medical records and data input were performed by trained coordinators at each participating center. The steering committee members of the Korean Sepsis Alliance conducted weekly audits and feedback to ensure data integrity. Any errors or discrepancies in the data were resolved through discussion with the investigators at each hospital with the steering committee.

© 2024 Ahn YH et al. *JAMA Network Open.*

eMethods 2. Baseline Clinical Factors Adjusted in the Main Analysis

The primary outcome was assessed using logistic regression analysis adjusted for the following baseline clinical factors associated with obesity, SA-AKI, or mortality: age, sex, comorbidities, Charlson Comorbidity Index (CCI), clinical frailty scale (CFS), Simplified Acute Physiology Score III (SAPS III), sequential organ failure assessment (SOFA) score excluding the kidney system (nonkidney SOFA score); use of mechanical ventilation, transfusion, or the presence of septic shock on the day of ICU admission; use of corticosteroids, vasopressors, and nephrotoxic antibiotics including glycopeptides, aminoglycosides, amphotericin B, or colistin; primary site and type of infection; and laboratory values including lactate, hemoglobin, albumin, and C-reactive protein (CRP).

© 2024 Ahn YH et al. *JAMA Network Open.*

eFigure 1. Study Design

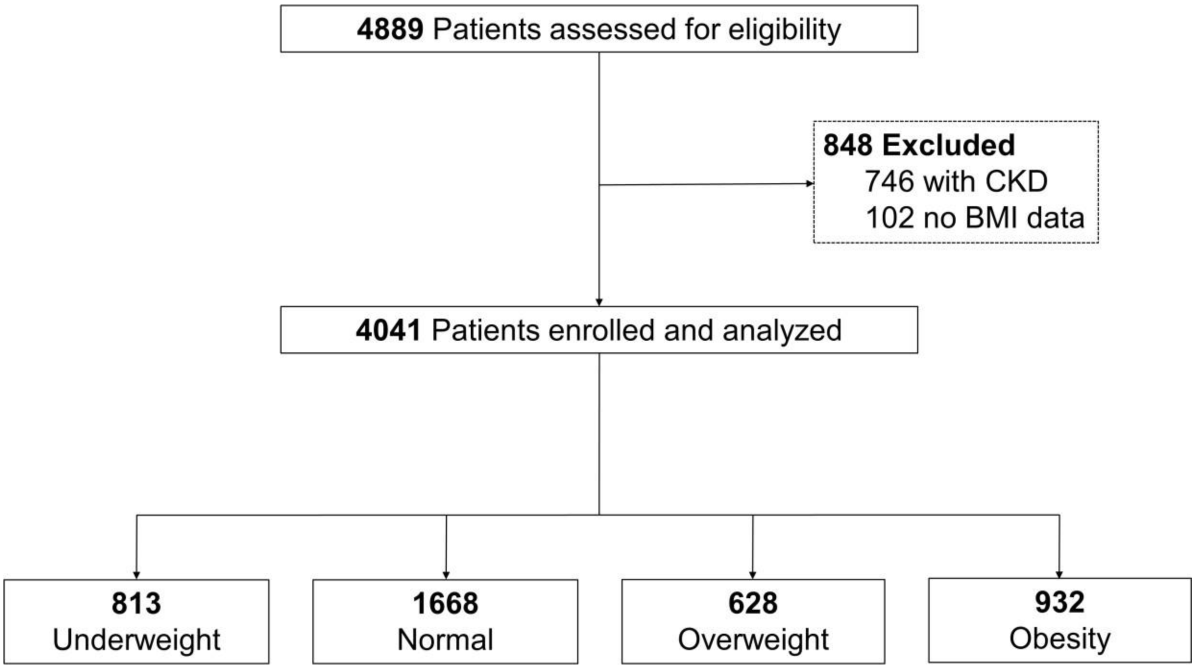

Abbreviations: BMI, body mass index; CKD, chronic kidney disease

© 2024 Ahn YH et al. *JAMA Network Open.*

eFigure 2. The Predicted Probability of Stage 3 Early SA-AKI

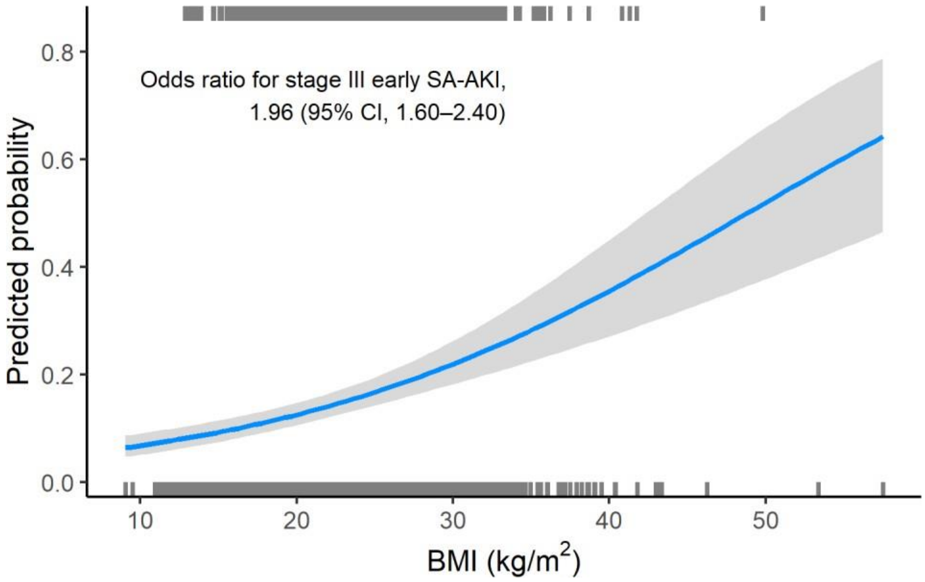

The probability of stage 3 early SA-AKI showed a linear increase with increasing BMI. A multivariable fractional polynomial regression model with a continuous BMI scale showed that every 10 kg/m^2 increase in BMI was associated with a 1.96 times higher risk of stage 3 early SA-AKI (OR, 1.96; 95% CI, 1.60–2.40). The shaded areas represent 95% confidence intervals. Abbreviations: AKI, acute kidney injury; BMI, body mass index; OR, odds ratio

© 2024 Ahn YH et al. *JAMA Network Open.*

eFigure 3. Association Between Body Mass Index and ICU Mortality

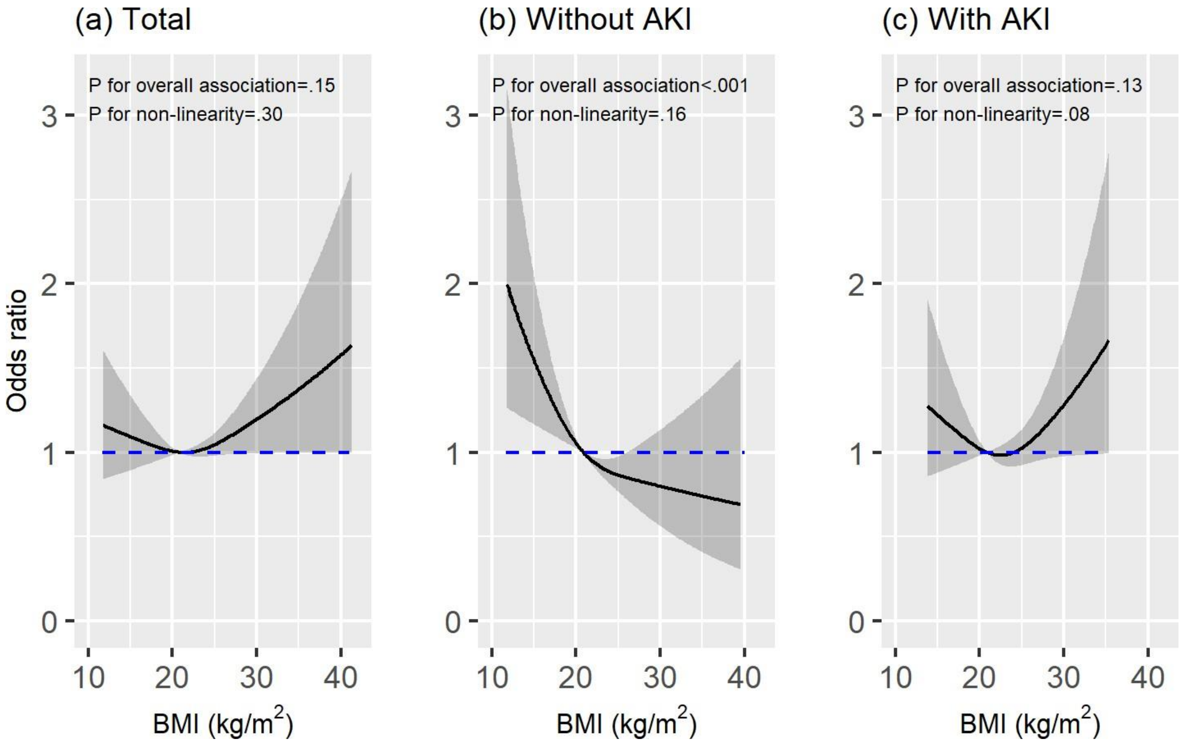

Panels a, b, and c show restricted cubic spline plots of the association between BMI and ICU mortality of the entire cohort, those without AKI, and those with AKI, respectively. The shaded areas represent 95% confidence intervals. The median BMI of the normal weight group (20.9 kg/m^2) was the reference standard, indicated by the dashed blue line. Abbreviations: AKI, acute kidney injury, BMI, body mass index.

© 2024 Ahn YH et al. *JAMA Network Open.*

eTable 1. Baseline Characteristics Across Body Mass Index Groups

Characteristic	Underweight (n = 813)	Normal weight (n = 1668)	Overweight (n = 628)	Obesity (n = 932)
Physiologic variables				
Mean Blood pressure, mmHg	78 ± 18	78 ± 19	79 ± 18	79 ± 19
Heart rate, beats/min	106 ± 24	107 ± 24	107 ± 24	106 ± 24
Respiratory rate, breaths/min	23 ± 7	23 ± 7	24 ± 7	24 ± 6
Body temperature, °C	36.8 ± 1.1	37.0 ± 1.0	37.2 ± 1.1	37.3 ± 1.1
Laboratory variables				
Bilirubin level, mg/dL	0.8 (0.5–1.3)	0.9 (0.6–1.7)	1.0 (0.6–2.0)	1.1 (0.6–2.2)
pH	7.37 ± 0.13	7.36 ± 0.13	7.36 ± 0.11	7.35 ± 0.12
$PaCO_2$, mmHg	36 ± 13	34 ± 11	33 ± 10	35 ± 11
PaO_2, mmHg	103 ± 50	103 ± 49	99 ± 43	98 ± 43
FiO_2, %	51 ± 25	50 ± 25	51 ± 24	51 ± 24
Nephrotoxic antimicrobials	153 (18.8)	411 (24.6)	165 (26.3)	260 (27.9)
Glycopeptide	139 (17.1)	377 (22.6)	156 (24.8)	242 (26.0)
Aminoglycoside	7 (0.9)	31 (1.9)	8 (1.3)	14 (1.5)
Colistin	12 (1.5)	12 (0.7)	3 (0.5)	7 (0.8)
Amphotericin B	0 (0)	6 (0.4)	4 (0.6)	6 (0.6)
Primary site of infection				
Pulmonary	506 (62.2)	751 (45.0)	246 (39.2)	312 (33.5)
Abdominal	172 (21.2)	471 (28.2)	205 (32.6)	328 (35.2)
Urinary	148 (18.2)	329 (19.7)	122 (19.4)	193 (20.7)
Catheter-related	5 (0.6)	21 (1.3)	9 (1.4)	11 (1.2)
Neurologic	5 (0.6)	13 (0.8)	4 (0.6)	8 (0.9)
Skin or soft tissue	18 (2.2)	70 (4.2)	25 (4.0)	51 (5.5)
Systemic infections without a primary site	35 (4.3)	145 (8.7)	61 (9.7)	100 (10.7)
Combination antibiotic therapy	504 (62.0)	1064 (63.8)	404 (64.3)	573 (61.5)
Pathogen identification	523 (64.3)	1100 (65.9)	427 (68.0)	612 (65.7)

Values are reported as n (%) for categorical variables and median (IQR) or mean ± SD for continuous variables.
Abbreviations: FiO_2, fraction of inspired oxygen; $PaCO_2$, partial pressure of carbon dioxide; PaO_2, partial pressure of oxygen

© 2024 Ahn YH et al. *JAMA Network Open.*

eTable 2. Baseline Characteristics of Patients With Missing Body Mass Index Data

Characteristic	Without BMI (n = 102)	With BMI (n = 4041)	*P*-value
Age, years	78 (65–83)	73 (63–81)	0.007
Sex			
Male	45 (44.1)	2349 (58.1)	0.006
Female	57 (55.9)	1692 (41.9)	
Comorbidities			
Cardiovascular disease	26 (25.5)	824 (20.4)	0.26
Diabetes	34 (33.3)	1400 (34.6)	0.87
Chronic lung disease	11 (10.8)	536 (13.3)	0.56
Chronic liver disease	5 (4.9)	395 (9.8)	0.14
Solid malignancy	11 (10.8)	1222 (30.2)	<0.001
Hematological malignancy	3 (2.9)	277 (6.9)	0.18
Chronic neurologic disease	30 (29.4)	984 (24.4)	0.29
Charlson comorbidity index	5 ± 2	5 ± 2	0.38
Clinical frailty scale	6 ± 2	5 ± 2	<0.001
SAPS III[a]	70 (63–82)	72 (62–84)	0.81
SOFA score[a]	8 (6–12)	9 (7–12)	0.41
Non-renal SOFA score[a]	8 (5–11)	9 (6–11)	0.24
Use of vasoactive medication[a]	66 (64.7)	3173 (78.5)	0.001
Mechanical ventilation[a]	36 (35.3)	1834 (45.4)	0.06
Transfusion[a]	20 (19.6)	905 (22.4)	0.58
Septic shock, n (%)[a]	46 (45.1)	2294 (56.8)	0.02
Creatinine level, mg/dl[a]	1.35 (0.90–2.10)	1.31 (0.84–2.11)	0.50
Lactate level, mmol/L[a]	2.3 (1.5–6.2)	2.8 (1.6–5.4)	0.63
Adjunctive corticosteroid therapy[a]	14 (13.7)	962 (23.8)	0.02
Primary site of infection, n (%)[a]			
Pulmonary	49 (48.0)	1815 (44.9)	0.60
Abdominal	22 (21.6)	1176 (29.1)	0.12
Urinary	26 (25.5)	792 (19.6)	0.18
Catheter-related	0 (0)	46 (1.1)	0.54
Neurologic	1 (1.0)	30 (0.7)	1.00
Skin or soft tissue	6 (5.9)	164 (4.1)	0.51
Systemic infections without a primary site	9 (8.8)	341 (8.4)	1.00
Type of infection			0.97
Community-acquired	62 (60.8)	2429 (60.1)	
Nosocomial	40 (39.2)	1612 (39.9)	
Multidrug resistant organism[a]	28 (27.5)	1061 (26.3)	0.88
Combination antibiotic therapy[a]	67 (65.7)	2545 (63.0)	0.65
Adequate antimicrobial therapy[a,b]	83 (81.4)	3535 (87.5)	0.09

Values are reported as *n* (%) for categorical variables and median (IQR) or mean ± SD for continuous variables.
Abbreviations: BUN, blood urea nitrogen; CRP, C-reactive protein; SAPS III, Simplified Acute Physiology Score III; SOFA, Sequential Organ Failure Assessment; WBC, white blood cell.
[a] On the day of ICU admission.
[b] Adequate empirical antimicrobial therapy was defined as the use of antibiotic agents with in vitro activity against suspected pathogens.

© 2024 Ahn YH et al. *JAMA Network Open.*

eTable 3. Early SA-AKI Incidence According to Body Mass Index, Including Severe Underweight and Obese Categories

Outcome	Severe underweight (n = 265)	Underweight (n = 813)	Normal weight (n = 1668)	Overweight (n = 628)	Obesity (n = 757)	Severe obesity (n = 175)
**Stage I or II or III SA-AKI**	49 (18.5)	152 (27.7)	546 (32.7)	242 (38.5)	296 (39.1)	82 (46.9)
Adjusted OR (95% CI)[a]	0.61 (0.42–0.87)[b]	0.78 (0.61–0.99)	1 (reference)	1.32 (1.06–1.63)	1.24 (1.02–1.52)	2.20 (1.53–3.15)[b]
**Stage III SA-AKI**	23 (8.7)	102 (18.6)	342 (20.5)	166 (26.4)	204 (26.9)	59 (33.7)
Adjusted OR (95% CI)[a]	0.45 (0.28–0.72)[b]	0.92 (0.70–1.20)	1 (reference)	1.40 (1.11–1.78)[b]	1.41 (1.13–1.76)[b]	2.37 (1.60–3.53)[b]

Abbreviations: AKI, acute kidney injury; CI, confidence interval; ICU, intensive care unit; OR, odds ratio
[a] Adjusted for age, sex, comorbidities, Charlson Comorbidity Index, clinical frailty scale, Simplified Acute Physiology Score III, non-renal sequential organ failure assessment score, mechanical ventilation, transfusion, septic shock, hemoglobin, lactate, albumin, C-reactive protein, use of vasopressors, corticosteroids, nephrotoxic antimicrobials, primary site of infection, and type of infection.
[b] Statistically significant after Bonferroni correction (*P*<0.01) compared with the normal weight group.

© 2024 Ahn YH et al. *JAMA Network Open.*

eTable 4. Summary of Multivariable Fractional Polynomial Transformations Applied to the Primary Outcome

Variables	In/Out of final model	FP	MFP transformation	*P*-value	OR (95% CI)
			**Stage I or II or III early SA-AKI**		
**Categorical**					
Sex	Out				
Cardiovascular disease	Out				
Chronic lung disease	Out				
Chronic neurologic disease	In	N/A		<0.001	0.73 (0.60–0.88)
Chronic liver disease	Out				
Diabetes	In	N/A		<0.001	1.43 (1.22–1.69)
Malignancy	In	N/A		0.004	0.76 (0.63–0.92)
Type of infection	Out				
Urinary infection	In	N/A		<0.001	1.49 (1.22–1.82)
Nephrotoxic antimicrobials	In	N/A		0.003	1.31 (1.10–1.56)
Mechanical ventilation	In	N/A		<0.001	1.50 (1.26–1.80)
Vasopressors	In	N/A		0.04	0.78 (0.62–0.99)
Septic shock	Out				
Adjunctive corticosteroid therapy	Out				
Transfusion	Out				
**Continuous**					
Age	Out				
BMI, kg/m^2	In	linear	BMI/10	<0.001	1.75 (1.47–2.08)
Charlson Comorbidity Index	In	linear	(Charlson Comorbidity Index + 1)/10	<0.001	0.51 (0.35–0.76)
Clinical frailty scale	Out				
SAPS III score	In	FP1	(SAPS III score/100)$^{-1}$	<0.001	0.14 (0.10–0.21)
Non-renal SOFA score	In	linear	(Non-renal SOFA score +1)/10	0.02	1.51 (1.08–2.11)
Lactate level, mmol/L	In	linear	Lactate/10	<0.001	5.10 (3.78–6.88)
		FP1	(Lactate/10)^{3}	<0.001	0.86 (0.80–0.92)
Hb, g/dL	In	FP1	(Hb/10)$^{-2}$	0.001	1.76 (1.24–2.49)
		FP1	(Hb/10)$^{-2}$ x log(Hb/10)	0.005	1.74 (1.19–2.54)
Albumin, g/dL	Out				
CRP, mg/dL	In	FP1	(CRP/10)^{3}	0.02	1.01 (1.00–1.01)

© 2024 Ahn YH et al. *JAMA Network Open.*

Stage III early SA-AKI					
**Categorical**					
Sex	Out				
Cardiovascular disease	Out				
Chronic lung disease	Out				
Chronic neurologic disease	In	N/A		<0.001	0.63 (0.50–0.79)
Chronic liver disease	Out				
Diabetes	Out				
Malignancy	In	N/A		<0.001	0.56 (0.46–0.69)
Type of infection	Out				
Urinary infection	In	N/A		<0.001	1.75 (1.40–2.19)
Nephrotoxic antimicrobials	In	N/A		<0.001	1.41 (1.16–1.73)
Mechanical ventilation	Out				
Vasopressors	In	N/A		0.01	0.71 (0.54–0.92)
Septic shock	Out				
Adjunctive corticosteroid therapy	In	N/A		0.005	1.32 (1.09–1.61)
Transfusion	Out				
**Continuous**					
Age	In	linear	Age/100	<0.001	0.23 (0.11–0.47)
BMI, kg/m^2	In	linear	BMI/10	<0.001	1.96 (1.60–2.40)
Charlson Comorbidity Index	Out				
Clinical frailty scale	In	FP1	(Clinical frailty scale/10)$^{-2}$	0.003	0.99 (0.99–1.00)
SAPS III score	In	FP1	(SAPS III score/100)$^{-2}$	<0.001	0.42 (0.35–0.49)
Non-renal SOFA score	In	FP1	((Non-renal SOFA score +1)/10)^{3}	<0.001	1.64 (1.07–1.27)
Lactate level, mmol/L	In	FP1	(Lactate/10)^{2}	<0.001	4.42 (3.51–5.57)
		FP1	(Lactate/10)^{2} x log(lactate/10)	<0.001	0.27 (0.20–0.35)
Hb, g/dL	In	FP1	(Hb/10)$^{-2}$	<0.001	2.65 (1.64–4.28)
		FP1	(Hb/10)$^{-2}$ x log(Hb/10)	<0.001	2.85 (1.59–5.09)
Albumin, g/dL	Out				
CRP, mg/dL	In	linear	CRP/10	0.02	1.12 (1.03–1.21)

Abbreviations: BMI, body mass index; CI, confidence interval; CRP, C-reactive protein; Hb, hemoglobin; OR, odds ratio; SAPS III, Simplified Acute Physiology Score III; SA-AKI, sepsis-associated acute kidney injury; SOFA, Sequential Organ Failure Assessment

© 2024 Ahn YH et al. *JAMA Network Open.*

eTable 5. Clinical Outcomes of Early SA-AKI According to Body Mass Index

Outcome	Underweight (n = 813)	Normal weight (n = 1668)	Overweight (n = 628)	Obesity (n = 932)	*P*-value[a]
Clinical recovery within 30 days[b,c]	366 (45.0)	818 (49.0)	313 (49.8)	480 (51.5)	0.05
ICU LOS, days[d]	5 (2–10)	4 (2–10)	5 (2–10)	4 (2–10)	0.37
Hospital LOS, days[d]	15 (7–28)	15 (8–28)	16 (8–28)	14 (7–25)	0.25
ICU mortality[b]	212 (26.1)	385 (23.1)	141 (22.5)	242 (26.0)	0.15
In-hospital mortality[b]	301 (37.0)	555 (33.3)	202 (32.2)	309 (33.2)	0.18
Discharge to home[b,e]	209/512 (40.8)[f]	640/1113 (57.5)	287/426 (67.4)[f]	449/623 (72.1)[f]	<0.001

Values are reported as *n* (%) for categorical variables and median (IQR) for continuous variables.
Abbreviations: CI, confidence interval; ICU, intensive care unit; LOS, length of stay; OR, odds ratio; SA-AKI, sepsis-associated acute kidney injury;
[a] Threshold for statistical significance following adjustment for multiple comparisons is *P*<0.017.
[b] Chi-square test of independence
[c] Clinical recovery was defined as survival to discharge within 30 days.
[d] Kruskal–Wallis test
[e] Only in patients who survived to discharge.
[f] Statistically significant after Bonferroni correction (*P*<0.017) compared with the normal weight group.

© 2024 Ahn YH et al. *JAMA Network Open.*

eTable 6. Clinical Outcomes According to Body Mass Index, Including Severe Underweight and Obese Categories

Outcome	AKI status	Severe underweight (n = 265)	Underweight (n = 813)	Normal weight (n = 1668)	Overweight (n = 628)	Obesity (n = 757)	Severe obesity (n = 175)
**ICU mortality**		59 (22.3)	153 (27.9)	385 (23.1)	141 (22.5)	189 (25.0)	53 (30.3)
Adjusted OR (95% CI)[a]	Without AKI	1.46 (0.92–2.33)	1.61 (1.14–2.28)[b]	1 (reference)	0.80 (0.53–1.22)	0.91 (0.62–1.35)	1.98 (0.93–4.21)
	With AKI	1.34 (0.69–2.62)	1.13 (0.76–1.69)	1 (reference)	0.99 (0.70–1.40)	1.11 (0.80–1.54)	1.61 (0.93–2.77)
**In-hospital mortality**		88 (33.2)	213 (38.9)	555 (33.3)	202 (32.2)	243 (32.1)	66 (37.7)
Adjusted OR (95% CI)[a]	Without AKI	1.32 (0.91–1.92)	1.44 (1.10–1.93)	1 (reference)	0.85 (0.62–1.16)	0.71 (0.52–0.98)	1.32 (0.70–2.48)
	With AKI	1.38 (0.71–2.66)	1.24 (0.84–1.85)	1 (reference)	0.92 (0.66–1.29)	1.10 (0.80–1.51)	1.73 (1.01–2.96)

Abbreviations: AKI, acute kidney injury; CI, confidence interval; ICU, intensive care unit; OR, odds ratio
[a] Adjusted for age, sex, comorbidities, Charlson Comorbidity Index, clinical frailty scale, Simplified Acute Physiology Score III, non-renal sequential organ failure assessment score, mechanical ventilation, transfusion, septic shock, hemoglobin, lactate, albumin, C-reactive protein, use of vasopressors, corticosteroids, nephrotoxic antimicrobials, primary site of infection, and type of infection.
[b] Statistically significant after Bonferroni correction (*P*<0.01) compared with the normal weight group.

© 2024 Ahn YH et al. *JAMA Network Open.*

eTable 7. Summary of Multivariable Fractional Polynomial Transformations Applied to the Secondary Outcome of In-Hospital Mortality

Variables	In/Out of final model	FP	MFP transformation	*P*-value	OR (95% CI)
			**In-hospital mortality (Total)**		
**Categorical**					
Sex	Out				
Cardiovascular disease	Out				
Chronic lung disease	Out				
Chronic neurologic disease	Out				
Chronic liver disease	Out				
Diabetes	Out				
Malignancy	Out				
Type of infection	In	N/A		0.006	0.80 (0.69–0.94)
Urinary infection	In	N/A		<0.001	0.62 (0.50–0.76)
Nephrotoxic antimicrobials	Out				
Mechanical ventilation	Out				
Vasopressors	In	N/A		<0.001	0.48 (0.38–0.59)
Septic shock	Out				
Adjunctive corticosteroid therapy	Out				
Transfusion	Out				
**Continuous**					
Age	Out				
BMI, kg/m^2	Out				
Charlson Comorbidity Index	Out				
Clinical frailty scale	In	linear	Clinical frailty scale/10	<0.001	3.01 (2.10–4.33)
SAPS III score	In	FP1	(SAPS III score)/100)$^{-1}$	<0.001	0.17 (0.12–0.24)
Non-renal SOFA score	In	FP1	((Non-renal SOFA score +1)/10)^2	<0.001	1.85 (1.59–2.15)
Lactate level, mmol/L	In	linear	Lactate/10	<0.001	3.12 (2.54–3.84)
Hb, g/dL	In	linear	Hb/10	0.004	0.59 (0.41–0.85)
Albumin, g/dL	In	linear	Albumin	<0.001	0.59 (0.51–0.68)
CRP, mg/dL	Out				

© 2024 Ahn YH et al. *JAMA Network Open.*

**In-hospital mortality (without SA-AKI)**					
**Categorical**					
Sex	Out				
Cardiovascular disease	Out				
Chronic lung disease	Out				
Chronic neurologic disease	Out				
Chronic liver disease	Out				
Diabetes	Out				
Malignancy	In	N/A		<0.001	1.68 (1.33–2.11)
Type of infection	In	N/A		<0.001	0.67 (0.54–0.83)
Urinary infection	In	N/A		<0.002	0.62 (0.46–0.84)
Nephrotoxic antimicrobials	Out				
Mechanical ventilation	Out				
Vasopressors	In	N/A		<0.001	0.39 (0.29–0.52)
Septic shock	Out				
Adjunctive corticosteroid therapy	Out				
Transfusion	Out				
**Continuous**					
Age	In	linear	Age/100	0.01	3.08 (1.28–7.40)
BMI, kg/m^2	In	linear	BMI/10	0.003	0.68 (0.52–0.88)
Charlson Comorbidity Index	Out				
Clinical frailty scale	In	linear	Clinical frailty scale/10	<0.001	3.65 (2.15–6.19)
SAPS III score	In	linear	SAPS III score/100	<0.001	10.11 (3.87–26.37)
Non-renal SOFA score	In	linear	(Non-renal SOFA score +1)/10	<0.001	3.85 (2.36–6.28)
Lactate level, mmol/L	In	linear	Lactate/10	<0.001	3.08 (2.24–4.25)
Hb, g/dL	Out				
Albumin, g/dL	In	linear	Albumin	<0.001	0.51 (0.42–0.62)
CRP, mg/dL	Out				
**In-hospital mortality (with SA-AKI)**					
**Categorical**					
Sex	Out				
Cardiovascular disease	Out				
Chronic lung disease	Out				
Chronic neurologic disease	Out				
Chronic liver disease	Out				
Diabetes	In	N/A		0.004	0.70 (0.55–0.89)

© 2024 Ahn YH et al. *JAMA Network Open.*

Malignancy	Out				
Type of infection	Out				
Urinary infection	In	N/A		<0.001	0.56 (0.41–0.76)
Nephrotoxic antimicrobials	Out				
Mechanical ventilation	Out				
Vasopressors	In	N/A		0.005	0.56 (0.38–0.84)
Septic shock	Out				
Adjunctive corticosteroid therapy	Out				
Transfusion	Out				
**Continuous**					
Age	Out				
BMI, kg/m^2	Out				
Charlson Comorbidity Index	Out				
Clinical frailty scale	In	linear	Clinical frailty scale/10	<0.001	3.07 (1.74–5.42)
SAPS III score	In	linear	SAPS III score/100	<0.001	8.68 (3.16–23.85)
Non-renal SOFA score	In	linear	(Non-renal SOFA score +1)/10	<0.001	3.39 (2.05–5.60)
Lactate level, mmol/L	In	linear	Lactate/10	<0.001	2.33 (1.77–3.08)
Hb, g/dL	Out				
Albumin, g/dL	In	linear	Albumin	<0.001	0.66 (0.54–0.81)
CRP, mg/dL	Out				

Abbreviations: BMI, body mass index; CI, confidence interval; CRP, C-reactive protein; Hb, hemoglobin; OR, odds ratio; SAPS III, Simplified Acute Physiology Score III; SA-AKI, sepsis-associated acute kidney injury SOFA, Sequential Organ Failure Assessment

© 2024 Ahn YH et al. *JAMA Network Open.*

eTable 8. ICU and In-Hospital Mortality Within Each Body Mass Index Category According to Early SA-AKI Status

	Underweight (n = 813)	Normal weight (n = 1668)	Overweight (n = 628)	Obesity (n = 932)
**ICU mortality**	212 (26.1%)	385 (23.1%)	141 (22.5%)	242 (26%)
Without SA-AKI	117/612 (19.1%)	141/1112 (12.6%)	41/386 (10.6%)	59/554 (10.6%)
With SA-AKI	95/201 (47.3%)	244/546 (44.7%)	100/242 (41.3%)	183/378 (48.4%)
Adjusted OR (95% CI)[a]	1.90 (1.19–3.01)[b]	3.05 (2.24–4.15)[b]	4.29 (2.47–7.45)[b]	4.30 (2.83–6.56)[b]
**In-hospital mortality**	301 (37.0%)	555 (33.3%)	202 (32.2%)	309 (33.2%)
Without SA-AKI	181/612 (29.6%)	255/1112 (22.7%)	83/386 (21.5%)	92/554 (16.6%)
With SA-AKI	120/201 (59.7%)	300/546 (54.9%)	119/242 (49.2%)	217/378 (57.4%)
Adjusted OR (95% CI)[a]	2.30 (1.50–3.53)[b]	2.47 (1.87–3.26)[b]	2.57 (1.61–4.08)[b]	4.22 (2.86–6.22)[b]

Abbreviations: CI, confidence interval; ICU, intensive care unit; OR, odds ratio; SA-AKI, sepsis-associated acute kidney injury

[a] Adjusted for age, sex, comorbidities, Charlson Comorbidity Index, clinical frailty scale, Simplified Acute Physiology Score III, non-renal sequential organ failure assessment score, mechanical ventilation, transfusion, septic shock, hemoglobin, lactate, albumin, C-reactive protein, use of vasopressors, corticosteroids, nephrotoxic antimicrobials, primary site of infection, and type of infection.

[b] *P*-values for comparisons between patients with SA-AKI and those without SA-AKI are statistically significant after Bonferroni correction (P<0.012)

© 2024 Ahn YH et al. *JAMA Network Open.*

eTable 9. Early SA-AKI Incidence and Clinical Outcomes of the Full Cohort, Including Excluded Patients With CKD

Variable / Outcome	Underweight (n = 936)	Normal weight (n = 1954)	Overweight (n = 771)	Obesity (n = 1126)	*P*-value[a]
Chronic kidney disease	123 (13.1)	286 (14.6)	143 (18.5)[g]	194 (17.2)	0.005
**Stage I or II or III SA-AKI**	284 (30.3)	736 (37.7)	339 (44.0)	510 (45.3)	
Adjusted OR (95% CI)[b]	0.76 (0.63–0.92)[g]	1 (reference)	1.25 (1.03–1.52)	1.32 (1.11–1.57)[g]	
**Stage III SA-AKI**	198 (21.2)	506 (25.9)	252 (32.7)	376 (33.4)	
Adjusted OR (95% CI)[b]	0.83 (0.66–1.03)	1 (reference)	1.37 (1.10–1.70)[g]	1.38 (1.14–1.68)[g]	
Clinical recovery within 30 days[c,d]	412 (44.0)[g]	958 (49.0)	384 (49.8)	570 (50.6)	0.016
ICU LOS, days[e]	5 (2–11)	4 (2–9)	5 (2–10)	4 (2–10)	0.15
Hospital LOS, days[e]	16 (7–29)	15 (8–28)	16 (8–28)	14 (8–25)	0.23
ICU mortality[c]	249 (26.6)	455 (23.3)	176 (22.8)	301 (26.7)	0.05
In-hospital mortality[c]	360 (38.5)	656 (33.6)	250 (32.4)	395 (35.1)	0.03
Discharge to home[c,f]	240/576 (41.7)[g]	761/1298 (58.6)	345/521 (66.2)[g]	522/731 (71.4)[g]	<0.001

Values are reported as *n* (%) for categorical variables and median (IQR) for continuous variables.
Abbreviations: CI, confidence interval; CKD, chronic kidney disease; ICU, intensive care unit; LOS, length of stay; OR, odds ratio; SA-AKI, sepsis-associated acute kidney injury
[a] Threshold for statistical significance following adjustment for multiple comparisons is *P*<0.017.
[b] Adjusted for age, sex, comorbidities, Charlson Comorbidity Index, clinical frailty scale, Simplified Acute Physiology Score III, non-renal sequential organ failure assessment score, mechanical ventilation, transfusion, septic shock, hemoglobin, lactate, albumin, C-reactive protein, use of vasopressors, corticosteroids, nephrotoxic antimicrobials, primary site of infection, and type of infection.
[c] Chi-square test of independence
[d] Clinical recovery was defined as survival to discharge within 30 days.
[e] Kruskal–Wallis test
[f] Only in patients who survived to discharge.
[g] Statistically significant after Bonferroni correction (*P*<0.017) compared with the normal weight group.

© 2024 Ahn YH et al. *JAMA Network Open.*
