# Supplementary material for: Early Sepsis-Associated Acute Kidney Injury and Obesity
Source: JAMA Netw Open. 2024 Feb 6;7(2):e2354923. doi: 10.1001/jamanetworkopen.2023.54923 (PMC10848068; doi:10.1001/jamanetworkopen.2023.54923)
Supplement: Supplement 2. — Korean Sepsis Alliance Investigators [file jamanetwopen-e2354923-s002.pdf]

Supplemental Online Content: Nonauthor Collaborators
*First name, last name, and suffix (if applicable) are required and will appear in PubMed.

*Group Name(s): Korean Sepsis Alliance Investigators							
***First Name and Middle Initial(s)**	***Last Name**	***Suffix (eg, Jr, III)**	Academic Degrees	Institution	Location (city, state/province, country)	Role or Contribution, eg, chair, principal investigator	Group (if more than 1 Group listed in the byline) and/or Subgroup (eg, Steering Committee)
Jeongwon	Heo		MD	Kangwon National University Hospital	Chuncheon, Gangwon-Do, Republic of Korea	Principal investigator	
Jae-myeong	Lee		PhD	Korea University Anam Hospital	Seoul, Seoul, Republic of Korea	Principal investigator	
Kyung Chan	Kim		PhD	Daegu Catholic University Hospital	Daegu, Daegu, Republic of Korea	Principal investigator	
Yeon Joo	Lee		PhD	Seoul National University Bundang Hospital	Seongnam, Gyeonggi-do, Republic of Korea	Principal investigator	Steering Committee
Young-Jae	Cho		PhD	Seoul National University Bundang Hospital	Seongnam, Gyeonggi-do, Republic of Korea	Principal investigator	Steering Committee
Sung Yoon	Lim		PhD	Seoul National University Bundang Hospital	Seongnam, Gyeonggi-do, Republic of Korea	Principal investigator	Steering Committee
Youjin	Chang		PhD	Inje University Sanggye Paik Hospital	Seoul, Seoul, Republic of Korea	Principal investigator	
Kyeongman	Jeon		PhD	Samsung Medical Center	Seoul, Seoul, Republic of Korea	Principal investigator	Steering Committee
Ryoung-Eun	Ko		PhD	Samsung Medical Center	Seoul, Seoul, Republic of Korea	Principal investigator	Steering Committee
Gee Young	Suh		PhD	Samsung Medical Center	Seoul, Seoul, Republic of Korea	Principal investigator	Steering Committee
Suk-Kyung	Hong		PhD	Asan Medical Center	Seoul, Seoul, Republic of Korea	Principal investigator	
Sang-Bum	Hong		PhD	Asan Medical Center	Seoul, Seoul, Republic of Korea	Principal investigator	Steering Committee
Woo Hyun	Cho		PhD	Pusan National University Yangsan Hospital	Yangsan, Gyeongsangnam-do, Republic of Korea	Principal investigator	
Sang Hyun	Kwak		PhD	Chonnam National University Hospital	Gwangju, Gwangju, Republic of Korea	Principal investigator	
Heung Bum	Lee		PhD	Jeonbuk National University Hospital	Jeonju, Jeollabuk-do, Republic of Korea	Principal investigator	
Jong-Joon	Ahn		PhD	Ulsan University Hospital	Ulsan, Ulsan, Republic of Korea	Principal investigator	
Gil Myeong	Seong		MD	Jeju National University Hospital	Jeju, Jeju, Republic of Korea	Principal investigator	
Song-I	Lee		PhD	Chungnam National University Hospital	Daejeon, Daejeon, Republic of Korea	Principal investigator	
Sunghoon	Park		PhD	Hallym University Sacred Heart Hospital	Anyang, Gyeonggi-do, Republic of Korea	Principal investigator	Steering Committee
Tai Sun	Park		PhD	Hanyang University Guri Hospital	Guri, Gyeonggi-do, Republic of Korea	Principal investigator	
Su Hwan	Lee		PhD	Severance Hospital	Seoul, Seoul, Republic of Korea	Principal investigator	
Eun Young	Choi		PhD	Yeungnam University Medical Center	Daegu, Daegu, Republic of Korea	Principal investigator	
Jae Young	Moon		PhD	Chungnam National University Sejong Hospital	Sejong, Sejong, Republic of Korea	Principal investigator	
Hyung Koo	Kang		MD	Inje University Ilsan Paik Hospital	Goyang, Gyeonggi-do, Republic of Korea	Principal investigator	
