# Supplementary material for: Early Sepsis-Associated Acute Kidney Injury and Obesity
Source: JAMA Netw Open. 2024 Feb 6;7(2):e2354923. doi: 10.1001/jamanetworkopen.2023.54923 (PMC10848068; doi:10.1001/jamanetworkopen.2023.54923)
Supplement: Supplement 3. — Data Sharing Statement [file jamanetwopen-e2354923-s003.pdf]

Data Sharing Statement

Ahn. Early Sepsis-Associated Acute Kidney Injury and Obesity. *JAMA Netw Open*. Published February 06, 2024. doi:10.1001/jamanetworkopen.2023.54923

Data
Data available: No

Additional Information
Explanation for why data not available: Dr. Hong Yeul Lee had full access to all the data in the study and takes responsibility for the integrity of the data and the accuracy of the data analysis. The datasets used and/or analyzed during the current study are available from the corresponding author on reasonable request.
